# Laser Promoting Oxygen Vacancies Generation in Alloy via Mo for HMF Electrochemical Oxidation

**DOI:** 10.1002/advs.202302641

**Published:** 2023-07-23

**Authors:** Junbo Liu, Shengyang Tao

**Affiliations:** ^1^ School of Chemistry Dalian University of Technology Dalian 116024 China; ^2^ State Key Laboratory of Fine Chemicals Dalian University of Technology Dalian 116024 China; ^3^ Frontier Science Center for Smart Materials Oriented Chemical Engineering Dalian University of Technology Dalian 116024 China

**Keywords:** HMF oxidation, laser processing, Mo doped, Ni alloy, oxygen vacancy

## Abstract

It is well known that nickel‐based catalysts have high electrocatalytic activity for the 5‐hydroxymethylfurfural oxidation reaction (HMFOR), and NiOOH is the main active component. However, the price of nickel and the catalyst's lifetime still need to be solved. In this work, NiOOH containing oxygen vacancies is formed on the surface of Ni alloy by UV laser (1J85‐laser). X‐ray absorption fine structure (XAFS) analyses indicate an interaction between Mo and Ni, which affects the coordination environment of Ni with oxygen. The chemical valence of Ni is between 0 and 2, indicating the generation of oxygen vacancies. Density functional theory (DFT) suggests that Mo can increase the defect energy and form more oxygen vacancies. In situ Raman electrochemical spectroscopy shows that Mo can promote the formation of NiOOH, thus enhancing the HMFOR activity. The 1J85‐laser electrode shows a longer electrocatalytic lifetime than Ni‐laser. After 15 cycles, the conversion of HMF is 95.92%.

## Graphical abstract

UV laser prepares a NiOOH electrode containing oxygen vacancies with long catalytic life for efficient electrooxidation of 5‐hydroxymethylfurfural. UV laser produces the generation of oxygen vacancies on the surface of the alloy. Mo doping can increase the vacancy formation energy and promote the formation of oxygen vacancies. Oxygen vacancies promote the oxidation of unsaturated coordination of Ni to NiOOH.

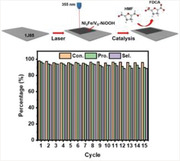

## 1 Introduction

2,5‐Furandicarboxylic acid (FDCA) is a high‐value‐added biomass derivative. The derivative of petroleum products, benzodicarboxylic acid, is an important raw material for producing plastic products.^[^ ^1^, ^2^, ^3^ ^]^ FDCA replacing benzodicarboxylic acid to produce biodegradable polyester can reduce white pollution and CO_2_ emissions.^[^ ^4^, ^5^, ^6^ ^]^ Compared to polyethylene terephthalate, a product of benzodicarboxylic acid polymerization, polyethylene furandicarboxylate offers significant advantages in terms of gas barrier properties.^[^ ^7^, ^8^ ^]^ The most promising synthetic method for FDCA is oxidative reaction from 5‐hydroxymethylfurfural (HMFOR). Compared to the harsh conditions of thermochemical syntheses, such as high temperature, high pressure, and precious metal catalysts. Electrocatalysis offers a more friendly route to synthesis.^[^ ^9^, ^10^, ^11^ ^]^

The activity of electrocatalytic reactions is mainly subject to the electrode material. The main electrode materials for HMFOR are oxides of transition metals, phosphides, sulfides, borides, and nitrides.^[^ ^11^, ^12^, ^13^, ^14^, ^15^, ^16^ ^]^ However, before using these electrode materials, the catalyst needs to be preoxidized using cyclic voltammetry (CV) to form a substable active phase on the electrode surface. Metal oxides have the advantages of stability and low toxicity, but the electron conduction of metal oxides is poor, and the main electroactive material is the hydroxyl oxide formed by oxidation on the catalyst surface.^[^ ^17^, ^18^, ^19^ ^]^ Introducing oxygen vacancies in metal oxides is a strategy to modulate the electronic structure,^[^ ^20^, ^21^, ^22^, ^23^ ^]^ which can enhance electron enrichment and promote the production of more hydroxy nickel oxide (NiOOH) from the unsaturated coordination of Ni.^[^ ^24^, ^25^, ^26^ ^]^ Common methods of introducing oxygen vacancies include heat treatment, doping, mechanical force, and laser ablation.^[^ ^27^, ^28^, ^29^, ^30^, ^31^, ^32^ ^]^ The advantages of laser processing include flexible processing, high energy density, and high processing efficiency. In addition, using the laser to process the electrode surface has less influence on the electrode surface's hot zone, and the electrode's deformation is small.^[^ ^30^, ^31^, ^32^, ^33^ ^]^

In this paper, we constructed a NiOOH electrode containing oxygen vacancies, with higher HMFOR activity and longer cycle life, by laser ablation on the surface of 1J85 (1J85‐laser electrode). The synchrotron X‐ray absorption fine structure (XAFS) analyses suggest the presence of coordination between Mo and Ni, which affects the coordination environment of Ni. The presence of unsaturated coordination between Ni and oxygen indicates the generation of oxygen vacancies. Density functional theory (DFT) calculations indicate that the presence of Mo increases the defect energy of the catalyst, which can generate more oxygen vacancies and accelerate electron transfer.^[^ ^34^, ^35^ ^]^ The study by in situ Raman electrochemical spectroscopy shows that Mo facilitates the formation of stable NiOOH on the surface of the electrode after laser processing, and the surface can be reconstructed to generate more NiOOH to improve the activity of HMFOR as the reaction proceeds.^[^ ^36^, ^37^ ^]^ Compared to the Ni‐laser electrode, the cycle life of the 1J85‐laser electrode was improved, maintaining a higher conversion of 92.91% for HMF and a higher yield of 87.47% for FDCA after 15 catalytic cycles. In addition, using electrocatalytic flow reactors can improve the mass transfer efficiency, enhance the activity of HMFOR, and shorten the reaction time. Notably, the Ni plate price is $60 per kilogram, while 1J85 alloy costs only $ 35.

## 2 Results and Discussion

### 2.1 Characterization of Catalytic Electrodes

**Scheme 1:**
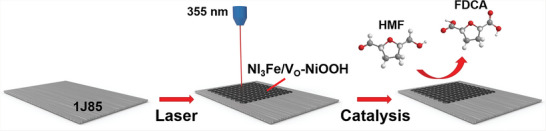
Schematic illustration of the preparation of 1J85‐laser electrode by a UV laser.

**Figure 1:**
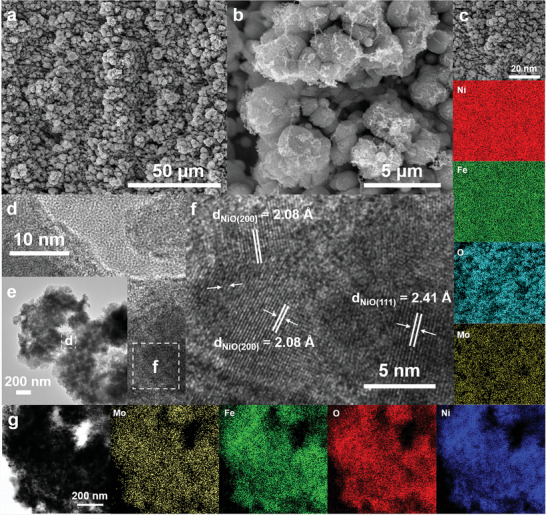
a,b) SEM images of 1J85‐laser surface. c) SEM image of 1J85‐laser and elemental mapping images. d,e) TEM images. f) HRTEM image. g) TEM elemental mapping images of 1J85‐laser electrode.

The preparation of the 1J85‐laser electrode and the electrocatalytic process of HMF are shown in **Scheme** **1**, and the details of the preparation of the 1J85 laser electrode are in the Experimental Section. The output current of the laser ranges from 31 to 39 A. The color of the surface changes after the laser ablates the electrode surface. When the laser current is small, the color of the electrode surface changes from bright silver to dark brown. The electrode color changes to light black when the laser current is higher. According to the above results, it is shown that the magnitude of the laser current has a significant effect on the formation of the oxide layer on the material surface. From the scanning electron microscope (SEM) images (Figure S1, Supporting Information), the material's surface was smooth before being laser ablated. After laser irradiation, a uniform oxide layer was formed. As the laser current increases, the oxide layer formed on the surface is gradually dense. The electrode surface prepared at 35 A current has a flocculent structure (**Figure** **1**a,b), which may increase the mass transfer capacity with the electrolyte. Figure 1c shows that the surface of the 1J85‐laser electrode is mainly composed of Ni, Fe, Mo, and O elements. Transmission electron microscopy (TEM) images reveal that the oxide layer on the electrode surface was composed of nanoparticles of oxides (Figure 1d,e,f shows the high‐resolution transmission electron microscopy (HRTEM) image, where the NiO (200) crystal plane with a lattice spacing of 2.08 nm and the (111) crystal plane with a lattice spacing of 2.41 nm can be found. Energy dispersive spectroscopy (EDS) mapping images show that the nanoparticles comprised uniform Ni, Fe, Mo, and O elements (Figure 1g).

**Figure 2:**
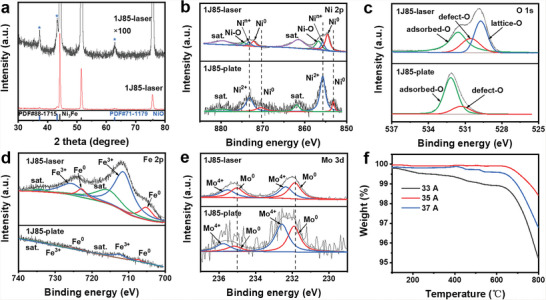
a) XRD pattern of 1J85‐laser electrode, the black line obtained by magnifying red line. b) HR XPS spectra of Ni 2p for 1J85 and 1J85‐laser. c) HR XPS spectra of Fe 2p. d) HR XPS spectra of Mo 3d. e) HR XPS spectra of O 1s. f) Thermogravimetric analysis of 1J85‐laser electrodes prepared at various laser currents.

The crystal structure was studied by X‐ray diffraction (XRD). The XRD pattern of 1J85 is shown in Figure S2 (Supporting Information). The diffraction peaks located at 44.17°, 51.44°, and 75.65° correspond to the (111), (200), and (220) crystal planes of Ni_3_Fe,^[^ ^38^ ^]^ respectively. The XRD pattern of the 1J85‐laser electrode shows that the (111) crystal plane of Ni_3_Fe was enhanced, and diffraction peaks attributed to NiO were observed (**Figure** **2**a). The diffraction peaks at 37.22 and 43.25 are attributed to the (111) and (200) crystal planes of NiO.^[^ ^39^ ^]^ The XRD results are consistent with the TEM images.

X‐ray photoelectron spectroscopy (XPS) (Figure S3, Supporting Information)) studies the elemental species, chemical bonding, and electronic structure on the electrode surface. The high‐resolution (HR) XPS spectrum of Ni 2p on the 1J85‐laser surface can be fitted to eight peaks, namely Ni^0^ (854.56/872.16 eV), Ni*^n^* ^+^ (855.71/873.31 eV), Ni–O (856.66/874.51 eV), and satellite peaks of Ni (Figure 2b).^[^ ^40^, ^41^, ^42^ ^]^ The presence of Ni*^n^* ^+^ indicates that the electrode surface formed unsaturated coordination sites between Ni and O after high‐energy laser irradiation, demonstrating the formation of oxygen vacancies. Ni–O indicates the formation of a stable NiOOH structure on the electrode surface. In contrast, only Ni^2+^ species and Ni^0^ of the alloy phase were present on the surface of the 1J85 alloy. The HR XPS spectrum of O 1s of the 1J85‐laser electrode can be divided into three characteristic peaks corresponding to lattice oxygen (529.76 eV), defective oxygen (530.56 eV), and adsorbed oxygen (531.61 eV) (Figure 2c).^[^ ^42^, ^43^, ^44^ ^]^ The presence of defective oxygen was caused by insufficient oxidation of Ni species during laser ablation, which also proved the existence of oxygen vacancies. Laser ablation can rapidly generate high temperatures with ultra‐fast heating and cooling rates, which facilitates defect formation.^[^ ^32^ ^]^ Only oxygen adsorbed on the material surface and a small amount of defective oxygen were observed in the O 1s spectrum of 1J85. Oxygen defects may come from the casting process of the 1J85 plate. The Fe 2p XPS spectrum of the 1J85‐laser electrode can be divided into six characteristic peaks corresponding to Fe^0^, Fe^3+^, and Fe satellite peaks (Figure 2d).^[^ ^38^, ^41^ ^]^ The Mo 3d XPS spectrum of the 1J85‐laser electrode can be divided into Mo^4+^ and Mo^0^ of the alloy phase (Figure 2e).^[^ ^40^, ^45^, ^46^ ^]^ The intensity of Fe 2p and Mo 3d on the surface of the 1J85 plate is very weak. Characteristic peaks of Mo^0^ and Fe^0^ belonging to the alloy and peaks of Fe^3+^ and Mo^4+^ caused by the oxidation of oxygen are observed. After laser ablation of the 1J85 surface in air, the intensity of Fe 2p and Mo 3d was significantly enhanced. Fe^3+^ and Mo^4+^ were formed by the oxidation of 1J85 with laser ablation, indicating the formation of a large number of oxides on the 1J85‐laser surface. The presence of Fe elements may improve the stability of NiOOH.^[^ ^40^ ^]^ In addition. the oxidation of low‐valent Ni to high‐valent Ni may be facilitated when Fe ions are adsorbed on the 1J85‐laser surface, which promotes the generation of NiOOH.^[^ ^47^ ^]^

Further, the XPS of the electrodes prepared at various laser output currents were investigated. The intensity of the characteristic peaks of defective oxygen and Ni^n+^ was stronger for the 35 A sample than the other samples, indicating that the 35 A sample contains more oxygen vacancies (Figure S4, Supporting Information). Figure 2f shows that the 35 A sample has the mildest weight loss. Because of the presence of oxygen vacancies, the oxygen vacancies were filled by oxygen during the heat treatment of thermal weight loss, resulting in a lower rate of weight loss.^[^ ^48^ ^]^ The results also show that the 35 A electrode has the highest oxygen vacancy content, consistent with the conclusion of XPS. Too high laser energy is not conducive to forming oxygen vacancies, probably because the excessive heat exacerbates the vaporization of the material surface.

**Figure 3:**
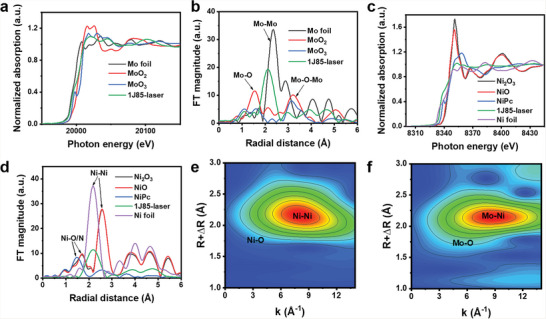
a) XANES spectra of Mo K‐edge. b) FT curves of the Mo K‐edge EXAFS. c) XANES spectra of Ni K‐edge. d) FT curves of the Ni K‐edge EXAFS. Wavelet transform of the Ni K‐edge e) and the Mo‐edge f) for 1J85‐laser.

Synchrotron X‐ray absorption fine structure (XAFS) were measured to further investigate the atomic coordination and electronic structure in the 1J85‐laser electrode. From the X‐ray absorption near‐edge structure (XANES) of Mo K‐edge (**Figure** **3**a), it can be obtained that the Mo valence in 1J85‐laser is higher than Mo Foil and lower than MoO_2_. The extended X‐ray

absorption fine structure (EXAFS) was characterized to scrutinize the coordination structure of the Mo‐related materials. In the Fourier transform (FT) EXAFS spectra of Mo K‐edge (Figure 3b), the 1J85‐laser has a distinct oscillation peak near 2.2 Å, located between Mo–Mo and Mo–O, which may be the result of coordination of Mo with other metals in the sample.^[^ ^49^ ^]^ The fitting results indicate that the coordination peaks of Mo are mainly composed of two pathways (Figure S5, Supporting Information), Mo–O and Mo–Ni, and the corresponding fitting parameters are shown in Table S1 (Supporting Information). The coordination of Mo with Ni affects the coordination environment of Ni, which suggests that the presence of Mo may promote the unsaturated coordination of Ni with O. XANES of Ni K‐edge shows that the Ni valence in 1J85‐laser is between 0 and +2 (Figure 3c), which indicates that the presence of unsaturated coordination of Ni with O leads to the generation of oxygen vacancies. The above results are consistent with the XPS conclusions. In the FT spectra of Ni K‐edge EXAFS (Figure 3d), the 1J85‐laser has a distinct oscillation peak near 2.2 Å, which may be a Ni–Ni metal bond. Compared to the oxide of Ni, the decrease in the coordination number of the Ni–Ni bond of 1J85‐laser is due to the lower filling of the 3d‐orbital of Ni in 1J85‐laser. The lower 3d‐orbital filling facilitates electrode binding to the reactants, enhancing electrocatalytic activity.^[^ ^50^ ^]^ According to the fitting results, the pathways of Ni coordination peaks are mainly composed of Ni–O and Ni–Ni. In the wavelet‐transformed EXAFS (WT‐EXAFS) plots (Figure 3e,f), the strong WT signal of the metal atom at the corresponding position in the R‐space can be observed. The signal of Ni/Mo–O is weaker in the WT‐EXAFS due to the lower coordination number. Reference diagrams for Ni and Mo foils are shown in Figure S6 (Supporting Information). The analysis of XAFS described above illustrates the coordination structure of Ni and Mo atoms in the 1J85‐laser electrode.

### 2.2 Electrochemical Characterization of Electrodes

**Figure 4:**
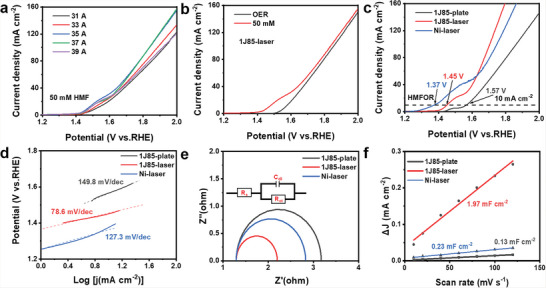
a) LSV curves of 1J85‐laser prepared at various laser currents. b) The LSV curves of 1J85‐laser in 1.0 m KOH with and without 50 × 10^−3^ m HMF. c) The LSV curves of 1J85 plate, 1J85‐laser, and Ni‐laser for HMFOR. d) Tafel slopes according to c). Nyquist curves e) and double‐layer capacitive currents f) of 1J85 plate, 1J85‐laser, and Ni‐laser, the inset of e) illustrate the equivalent circuit diagram.

The electrode's oxygen evolution reaction (OER) and HMFOR activity in 1.0 m KOH with and without 50 × 10^−3^ m HMF were investigated with linear sweep voltammetry (LSV). The LSV curves of the 1J85‐laser electrodes prepared at various UV laser currents indicate that the electrocatalytic activity first increases and then decreases with increasing current (**Figure** **4**a). The 35 A sample had the lowest OER activity with a reaction overpotential of 339 mV (Figure S7, Supporting Information), indicating that the OER activity was inhibited. When 50 × 10^−3^ m of HMF was added to the electrolyte, the electrocatalytic reaction's overpotential decreased, indicating that the electrode was more inclined to catalyze HMFOR (Figure 4b). The electrocatalytic performance of the electrodes before and after laser ablation was characterized, and the overpotential of HMFOR decreased from 1.57 to 1.45 V (Figure 4c). The above results indicate that the presence of oxygen vacancies adjusts the electronic state of the electrode and may generate more NiOOH,^[^ ^26^, ^51^ ^]^ which is the active site for HMF oxidation. Although the Ni‐laser electrode has a lower overpotential for the HMF oxidation reaction, its kinetic rate is lower than that of the 1J85‐laser electrode. The analysis of the exchange current density and Tafel slope shows that the 1J85‐laser electrode has a higher exchange current density (Figure S8, Supporting Information) and lower Tafel slope (Figure 4d), indicating its good kinetic performance.^[^ ^52^ ^]^ The electrochemical impedance spectrogram (EIS) is used to characterize the electron transfer rate of the electrode, and *R*_ct_ represents the charge transfer resistance in the equivalent circuit diagram.^[^ ^26^ ^]^ The 1J85‐laser electrode has the lowest *R*_ct_, indicating its better electron‐transfer performance. In addition, the HMFOR activity of the 1J85‐laser electrode was also better than most of the reported electrodes (Table S2, Supporting Information). The electrochemical active area (ECSA) of the electrodes was further investigated. The ECSA can be calculated from the double‐layer capacitance (*C*_dl_), and the *C*_dl_ values of the electrodes were obtained by CV (Figure S9, Supporting Information).^[^ ^18^ ^]^ The 35 A sample has the highest *C*_dl_ value (Figure 4f), indicating that it has the largest ECSA, leading to the highest HMFOR activity, consistent with the conclusions obtained by LSV.

For overall water splitting, using HMF oxidation for the anode instead of OER can reduce the voltage of the reaction system.^[^ ^53^ ^]^ Therefore, it is also essential to study the HER performance of the electrodes. The 1J85‐laser electrode exhibits better HER performance than the 1J85 plate (Figure S10, Supporting Information). The lower HER overpotential, the lower tafel slope, and the smaller *R*_ct_ value evidence this. The HER results also demonstrate the importance of oxygen vacancies for regulating electronic structure.

### 2.3 Product Analysis

**Figure 5:**
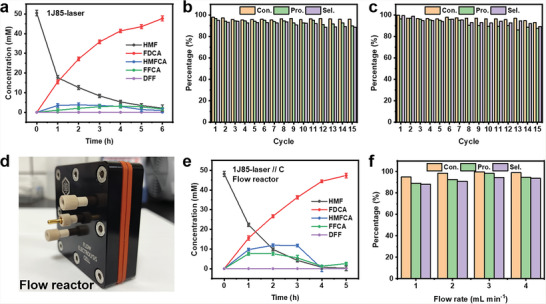
a) Curves of the concentrations of HMF, HMFCA, FFCA, FDCA, and DFF versus reaction time using 1J85‐laser. HMF conversion, FDCA selectivity, and FDCA productivity in 15 successive cycles with 1J85‐laser b) and Ni c). (d) Photo of the flow reactor. e) Curves of the concentrations of HMF, HMFCA, FFCA, FDCA, and DFF versus reaction time with flow reactor at an electrolyte flow rate of 3 mL min^−1^. f) Conversion of HMF, selectivity of FDCA, and FDCA productivity at various electrolyte flow rates with flow reactor.

The products of electrocatalytic oxidation of HMF were analyzed with HPLC. The external calibration curves of chemicals are shown in Figure S11 (Supporting Information). The 1J85‐laser electrode oxidized the HMF with an anodic voltage of 1.55 V (Figure S12, Supporting Information). With the increase in reaction time, HMF was gradually oxidized, and the products were mainly 5‐hydroxymethyl‐2‐furancarboxylic acid (HMFCA), 5‐formyl‐2‐furancarboxylic acid (FFCA), and FDCA (**Figure** **5**a and Figure S13, Supporting Information). 50 × 10^−3^ m of HMF was fully converted in a reaction time of 6 h (Figure S14, Supporting Information). Since no 2,5‐diformylfuran (DFF) was detected during the reaction, we assume that the oxidation pathway of HMF is shown in Figure S15 (Supporting Information).^[^ ^9^, ^10^, ^11^ ^]^ The reaction kinetics of HMF oxidation was investigated using reaction rate equations. Assuming that the reactions follow first‐order and pseudo‐first‐order kinetic model,^[^ ^53^ ^]^ the reaction rate constant k was obtained from the concentration of HMF oxidation products versus reaction time based on the data from LC. *k*_1_ (1.19 × 10^−2^ s^−1^) is much smaller than *k*_2_ (2.34 × 10^−1^ s^−1^) and *k*_3_ (5.15 × 10^−2^ s^−1^), which indicates that the electrooxidation of HMF to HMFCA is the decisive stage of the total reaction.

Several electrocatalysis experiments were performed with constant voltage electrocatalysis to study the cycle life of the electrodes. After 15 times catalytic reactions (Figure S16, Supporting Information), the 1J85‐laser electrode exhibited higher HMFOR activity than the Ni‐laser electrode. The conversion of HMF could be maintained at 95.92%, and the yield of FDCA was 89.71% (Figure 5b). While the conversion rate of the Ni‐laser electrode to HMF decreased to 92.91%, and the yield of FDCA decreased to 87.47% (Figure 5c). Based on the FE, it can also be concluded that the HMFOR activity of the 1J85‐laser electrode is greater (Figure S17, Supporting Information). After 5 times catalytic reactions, the FE of FDCA is 85.7% with 1J85‐laser electrode, and the FE with Ni‐laser is 81.7%.

XPS of the 1J85‐laser electrode after HMFOR can confirm the increased content of oxygen vacancies after the first catalytic reaction (Figure S18, Supporting Information). Because the intensity of the peak of defective oxygen is enhanced. For Ni, the enhanced relative intensity of the Ni^n+^ also suggests more unsaturated coordination of Ni with O. More oxygen vacancies make the electrode more favorable for the generation of NiOOH active sites.^[^ ^26^, ^51^ ^]^ The NiOOH generated during the reaction can improve the activity of HMFOR. For Mo and Fe, there were no significant changes in chemical valence. XRD analysis of the 1J85‐laser electrode after HMFOR revealed that the intensity of the diffraction peaks on the Ni_3_Fe (111) crystal plane decreased (Figure S19, Supporting Information), which could be attributed to the generation of more NiOOH after the first HMFOR. The diffraction streaks of NiOOH can be observed in the TEM of the 1J85‐laser electrode after the first HMFOR (Figure S20, Supporting Information), and an increase in the oxygen content is observed in the EDS of the TEM (Table S3, Supporting Information), which proves that more NiOOH is generated. Since more NiOOH was produced on the electrode surface, which increased the ECSA of the electrode, the *C*_dl_ value of the 1J85‐laser was 2.09 mF cm^−2^ after the first HMFOR (Figure S21, Supporting Information). More easily formed NiOOH may be why the 1J85‐laser electrode still maintains good electrocatalytic activity.

Further, the XPS spectra of the 1J85‐laser electrode after 15 catalytic cycles were studied (Figure S22, Supporting Information). The Ni and Fe on the electrode surface were oxidized to higher valence states. Ni^2+^ and Ni^3+^ can be observed in the Ni 2p XPS spectrum. The characteristic peak of Ni^n+^ disappeared, indicating the disappearance of oxygen vacancies. Fe 2p XPS spectrum shows that Fe0 on the electrode surface was oxidized to Fe^2+^ and Fe^3+^. The element Mo is relatively stable. It was no further oxidized. O 1s XPS spectrum can be divided into lattice oxygen bound to metals and adsorbed oxygen. C 1s XPS spectra can be divided into C–C, C–O, and C═O. The small amount of C–O (285.61 eV) and C═O (288.46 eV) on the 1J85‐laser surface before the reaction came from carbon pollution in the air. After the reaction, the 1J85‐laser was poisoned by the reaction intermediates and the intensity of the characteristic peaks of C–O and C═O was enhanced. Therefore, the decrease in the catalytic performance of the 1J85‐laser electrode may be due to the fact that the absence of oxygen vacancies cannot continue to promote the oxidation of Ni^n+^ to NiOOH, and NiOOH is continuously lost during the reaction. In addition, the 1J85‐laser electrode adsorb reaction intermediates during the reaction, leading to catalyst poisoning.

Mass transfer efficiency is one of the significant factors affecting electrocatalytic reactions. The flow electrocatalysis strategy can significantly improve mass transfer efficiency.^[^ ^54^ ^]^ Using a flow reactor (Figure 5d), 50 × 10^−3^ m of HMF can be converted in only 5 h. The 1J85‐laser and graphite plates were placed into the flow electrochemical reactor, acting as anode and cathode, separated by a Nafion 117 membrane (Figure S23, Supporting Information). The catalytic activity of HMFOR was investigated at various electrolyte flow rates, and the flow reactor exhibited the highest HMFOR activity when the flow rate was 3 mL min^−1^ (Figure 5e,f).

### 2.4 Catalytic Mechanism Analysis

**Figure 6:**
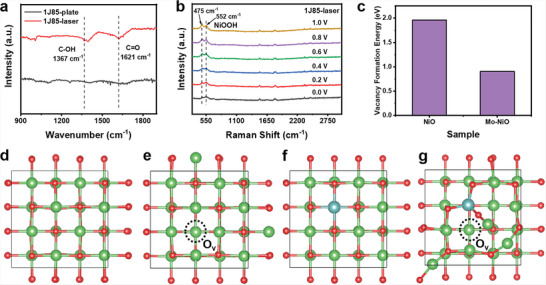
a) Infrared spectra of HMF on 1J85 plate and 1J85‐laser. b) In situ Raman electrochemical spectra of 1J85‐laser at various potentials in 1.0 m KOH with 50 × 10^−3^ m HMF. c) Oxygen vacancy formation energy of NiO with and without Mo doping. d,e) Oxygen vacancy model of NiO crystal plane (200). f,g) Oxygen vacancy model of Mo‐doped NiO crystal plane (200).

The adsorption state of HMF on the electrode surface was investigated using infrared (IR) spectroscopy. The C–OH absorption peak located at 1367 cm^−1^ and the C═O absorption peak at 1621 cm^−1^ can be observed in the IR spectrum of the 1J85‐laser electrode (**Figure** **6**a), which indicates that the surface of the laser electrode is more easily to adsorb HMF, facilitating the start of electrocatalytic reactions.^[^ ^11^, ^55^ ^]^ A mechanism of oxidation of HMF by a 1J85‐laser electrode is proposed, where Mo in the alloy material facilitates the generation of more oxygen vacancies during laser treatment, and unsaturated coordination of Ni promotes the generation of NiOOH. The Raman spectra showed that the 1J85‐laser electrode formed a stable NiOOH structure on the surface after laser ablation corresponding to a strong Raman peak at 560 cm^−1^ for the bending vibration and 684 cm^−1^ for the stretching vibration (Figure S24, Supporting Information).^[^ ^49^ ^]^ With in situ Raman electrochemical spectra, we investigated the 1J85‐laser electrode and the Mo‐free alloy materials 1J36 and 1J50, and the number represents the Ni content in the material. According to the results of previous studies, laser ablation of Ni plates does not directly produce stable NiOOH,^[^ ^56^ ^]^ which is obtained by reconstitution in the process of catalytic reaction. For 1J36 and 1J50 alloys with lower Ni content and without Mo, only Ni–O Raman absorption peaks were observed, and NiOOH absorption peaks did not appear as the reaction proceeded (Figure S25, Supporting Information). This conclusion was also evidenced by the fact that Ni‐O was not observed in the XPS spectra of 1J36‐laser and 1J50‐laser (Figure S4, Supporting Information). Further, we investigated the in‐situ Raman electrochemical spectra of the 1J85‐laser electrode, and the Raman absorption peaks of NiOOH located at 475 and 552 cm^−1^ were observed when the electrode voltage was 0 V (Figure 6b).^[^ ^57^ ^]^ This indicated the presence of stable NiOOH on the electrode surface before the beginning of HMFOR. And more NiOOH was produced as the reaction proceeded (Figure S26, Supporting Information).^[^ ^49^, ^56^ ^]^ The generated NiOOH indicates that oxygen vacancies can promote the reconstruction of the electrode surface. NiOOH is the active component of HMFOR and can improve its activity of HMFOR. Therefore, we believe that the presence of Mo can promote the electrode to generate more oxygen vacancies during laser ablation, and the oxygen vacancies promote the oxidation of surface Ni atoms to NiOOH, which leads to the improvement of HMFOR activity.

DFT calculations further verified the proposed mechanism. The doped and undoped Mo models were constructed on the (200) crystal plane of NiO (Figure 6d–g and Figure S27, Supporting Information). The calculations show that the Mo doping makes the material more prone to generate oxygen vacancies, and the vacancy formation energy is 0.906 eV, which is much smaller than the 1.960 eV of the undoped model (Figure 6c). The more positive vacancy formation energy indicates that the formation of vacancies is more complicated. Therefore, Mo doping makes the formation of oxygen vacancies easier.

## 3 Conclusion

In summary, we prepared NiOOH electrodes containing oxygen vacancies for electrocatalytic oxidation of HMF with a pulsed UV laser. Compared with the Ni‐laser electrode, the 1J85‐laser electrode has a longer catalytic cycle life and can be used as one of the ideal electrodes to replace the Ni‐laser electrode, solving the problem of the high cost of Ni plates. XAFS demonstrates that interaction between Mo and Ni leads to a change in the coordination environment of Ni, making the 1J85 alloy more prone to the formation of oxygen vacancies during laser ablation. The DFT calculations verify this conclusion that the presence of Mo can increase the defect energy of the electrode and thus make it easier to generate oxygen vacancies. In‐situ Raman electrochemical spectroscopy demonstrates that the presence of oxygen vacancies makes the electrode surface more susceptible to the formation of NiOOH. NiOOH, as the active site for electrocatalytic reactions, can enhance HMFOR activity. This work proposes an effective laser strategy to introduce oxygen vacancies and generate stable NiOOH structures for catalytic oxidation of HMF to generate FDCA.

## 4 Experimental Section

### Chemicals

Ni plate with a thickness of 0.1 mm and a purity of 99.99% was bought from the Shengshi Da Metal Materials Co. 1J36, 1J50, and 1J85 plates (thickness of 0.1 mm, purity of 99.99%) were bought from the Sajing Special Alloys Co. 1J35, 1J50, and 1J85 are Ni alloys, where the number represents the approximate Ni content. 1J85 plate (50 × 50 × 3 mm) was bought from the Shengyu Metal Materials Co. HMF (AR) was bought from Aladdin Shanghai Co. HMF, FDCA, HMFCA and FFCA (standard for LC) were also bought from Aladdin Shanghai Co. Potassium hydroxide (KOH, AR) was bought from Kemei Chemical Tianjin Co. The proton exchange membrane with electrical conductivity of 0.083 S cm^−1^ (Nafion 117) was bought from DuPont Co. All electrolyte solutions were prepared using ultra‐pure water. All materials were no further treatment.

### Laser Preparation of Electrodes

First, the surface of the metal sheets was gently sandpapered to remove the oxide layer, then ultra‐sonicated with ethanol and water three times, respectively, and finally blown dry with nitrogen gas and set aside.

For the preparation of Ni‐laser, 1J36‐laser and 1J50‐laser electrodes, the surface of metal electrodes was ablated with a nanosecond UV laser (UV‐3C) purchased from HAN'S LASER Co. The total power of the pulsed laser is 3 W, and the wavelength of the UV laser is 355 nm. The scanning speed of the laser spot was set to 100 mm s^−1^ and the laser current was 35 A. The electrodes were prepared with an area of 2 cm^2^ and then washed with deionized water.

For the preparation of 1J85 electrodes, the process was the same as above, except that the output current range was set to 31–39 A.

### Physical Characterization of Electrodes

The microstructure and energy dispersive spectroscopy (EDS) of the electrodes were studied by a scanning electron microscope (FEI QUANTA 450).TEM, HRTEM and TEM elemental mapping images were analyzed by a transmission electron microscope (HT7700 EXALENS). For the preparation of TEM samples, the oxide layer on the electrode surface is scraped off and placed in ethanol for ultrasonic treatment. Raman spectra of electrodes were investigated with a DXR Raman microscope with a laser wavelength of 532 nm (Renishaw inVia Qontor). In situ Raman electrochemical spectra were obtained by coupling a DXR Raman microscope with an electrochemical workstation (Chenhua CHI 660E). The working electrode (laser electrodes), counter electrode (platinum wire) and reference electrode (Ag/AgCl) were assembled into the in‐situ Raman spectroscopy test cell. A Fourier transform infrared spectrometer (Nicolet iS50) was used to study the infrared spectra of the electrodes. An X‐ray diffractometer (SmartLab 9 kW, Cu Kα radiation with 40 kV and 100 mA) was used to measure the X‐ray diffraction (XRD) patterns. An X‐ray photoelectron spectrometer (XPS, Thermo Scientific ESCALAB Xi+) analyzed the electrode surface XPS spectra

### Electrochemical Characterization of Electrodes

A three‐electrode system was used to research the electrocatalytic processes using Chenhua's electrochemical workstation at ambient temperature and pressure, the working electrodes were the prepared laser electrodes (electrode area of 2 cm^2^). A Platinum sheet and a Ag/AgCl electrode with saturated potassium chloride (KCl) solution were served as counter electrode and reference electrode, respectively. The electrocatalytic reaction was performed in an H‐type electrolytic cell separated by a Nafion 117 proton exchange membrane. HMFOR was performed under constant potential method with an anode electrode potential of 1.55 V (vs RHE). The electrolyte solution of 30 mL was magnetically stirred at 400 rpm. Prior to the start of the test, the working electrodes were subjected to 20 cycles of CV scanning in electrolyte solution. In addition, when using a flow reactor, the reaction time was 5 h. The anode electrolyte was a 1.0 m KOH solution containing 50 × 10^−3^ m HMF, and the cathode electrolyte was a 1.0 m KOH solution. Linear scanning voltammetry (LSV) was performed at a scanning rate of 5 mV s^−1^ and a potential range from 0.0 to 1.0 V. Cyclic voltammetry (CV) was performed at scanning rates from 20 to 120 mV s^−1^ with a potential range of −0.851 to −0.051 V. According to the Ag/AgCl (V)  =  RHE (V)  +  0.0592  ×  pH, the reference electrode potential was converted to reversible hydrogen electrode (RHE) potential. Electrochemical impedance spectroscopy (EIS) were obtained in the frequency range of 10^−2^ to 10^5^ Hz with 5 mV amplitude.

### Product Analysis

Quantitative and qualitative analysis of the oxidation products of HMF was performed with Agilent's high‐performance liquid chromatography (HPLC, 1260 Infinity II). During the constant potential electrocatalytic reaction, 100 µL of the sample was removed from the reaction solution at regular intervals, and the sample was diluted with 1000 µL of distilled water. HPLC quantified the diluted sample. The HPLC is equipped with a C18 column and a UV–Vis detector with a wavelength of 265 nm. The mobile phase was 5 × 10^−3^ m ammonium formate solution and methanol in the ratio of 7:3,^[^ ^19^ ^]^ the detection time was 4 min, the mobile phase flow rate was 1 mL min^−1^, and the sample injection volume was 5 µL. LC standards of the product were diluted and then injected into the HPLC for qualitative analysis.

The conversion rate of HMF (%), the productivity of FDCA (%), the selectivity of FDCA (%), and the Faradaic efficiency (FE) to FDCA (%) were calculated according to the following equations

(1) $$\mathrm{HMF}\;\mathrm{conversion}\;(\%)=\frac{\mathrm{mol}\;\mathrm{of}\;\mathrm{HMF}\;\mathrm{consumed}}{\mathrm{mol}\;\mathrm{of}\;\mathrm{initial}\;\mathrm{HMF}}\times 100\%$$

(2) $$\mathrm{FDCA}\;\mathrm{productivity}\;(\%)=\frac{\mathrm{mol}\;\mathrm{of}\;\mathrm{FDCA}\;\mathrm{formed}}{\mathrm{mol}\;\mathrm{of}\;\mathrm{initial}\;\mathrm{HMF}}\times 100\%$$

(3) $$\mathrm{FDCA}\;\mathrm{selectivity}\;(\%)=\frac{\mathrm{mol}\;\mathrm{of}\;\mathrm{FDCA}\;\mathrm{formed}}{\begin{array}{c} \mathrm{mol}\;\mathrm{of}\;\mathrm{products}\;\mathrm{formed}\\ \;+\;\mathrm{mol}\;\mathrm{of}\;\mathrm{HMF}\;\mathrm{unreacted} \end{array}}\times 100\%$$

(4) $$\mathrm{Faradaic}\;\mathrm{efficiency}\;(\%)=\mathrm{mol}\;\mathrm{of}\;\mathrm{FDCA}\;\mathrm{formed}\times \frac{nF}{It}\times 100\%$$

where *n* is the number of electrons transferred (*n* = 6 for the conversion of HMF to FDCA), *I* is the current gained in the HMFOR, *t* is the reaction time of the electrocatalysis experiment, and *F* is the Faraday's constant (96485 C mol^−1^).

## Conflict of Interest

The authors declare no conflict of interest.

## Supporting information

Supporting Information

## Data Availability

The data that support the findings of this study are available from the corresponding author upon reasonable request.
