# Supplementary material for: Laser Promoting Oxygen Vacancies Generation in Alloy via Mo for HMF Electrochemical Oxidation
Source: Adv Sci (Weinh). 2023 Jul 23;10(27):2302641. doi: 10.1002/advs.202302641 (PMC10520653; doi:10.1002/advs.202302641)
Supplement: Supplementary file 1 — Supporting Information [file ADVS-10-2302641-s001.pdf]

ADVANCED SCIENCE
Open Access

Supporting Information

for *Adv. Sci.*, DOI 10.1002/advs.202302641

Laser Promoting Oxygen Vacancies Generation in Alloy via Mo for HMF Electrochemical Oxidation

*Junbo Liu and Shengyang Tao**

Supporting Information

Laser promoting oxygen vacancies generation in alloy via Mo for HMF electrochemical oxidation

*Junbo Liu (c), and Shengyang Tao (a,b,c)**

[a] Shengyang Tao* State Key Laboratory of Fine Chemicals, Dalian University of Technology, Dalian 116024, China.

E-mail: taosy@dlut.edu.cn

[b] Shengyang Tao* Frontier Science Center for Smart Materials Oriented Chemical Engineering, Dalian University of Technology, Dalian 116024, China.

[c] Junbo Liu, Shengyang Tao* School of Chemistry, Dalian University of Technology, Dalian 116024, China.

Density functional theory (DFT)

We have employed the Vienna Ab initio Simulation Package (VASP) to perform all density functional theory (DFT) calculations. The projector represented the elemental core and valence electrons augmented wave (PAW) method and plane-wave basis functions with a cutoff energy of 450 eV. Generalized gradient approximation with the Perdew-Burke-Ernzerh of (GGA-PBE) exchange-correlation functional was employed in all the calculations. Geometry optimizations were performed with the force convergency smaller than 0.05 eV/Å. Mo-NiO (200) is one Mo atom doping the Ni atoms on the NiO (200) surface. Monkhorst-Pack k-points of 2×2×1 were applied for all the calculations. Half atoms at the bottom are fixed in all the calculations. We used the following equations to calculate the vacancy formation energy in that order.

$$\Delta E = E(O_v) + \frac{1}{2}E(O_2) - E(surf)$$

Here, $E(O_v)$ was the total energy of the vacancy surface. $E(O_2)$ was the energy of O_2. E_{surf} was the surface energy.

X-ray absorption fine structure (XAFS) analysis

Ni and Mo K-edge analysis were performed with Si (111) crystal monochromators at the BL14W1 beamlines at the Shanghai Synchrotron Radiation Facility (SSRF) (Shanghai, China). Before the analysis at the beamline, samples were pressed into thin sheets with 1 cm in diameter and sealed using Kapton tape film. The XAFS spectra were recorded at room temperature using a 4-channel Silicon Drift Detector (SDD) Bruker 5040. Ni and Mo *K*-edge extended X-ray absorption fine structure (EXAFS) spectra were recorded in transmission mode). Negligible changes in the line-shape and peak position of Ni and Mo *K*-edge XANES spectra were observed between two scans taken for a specific sample. The XAFS spectra of these standard samples (Ni foil, NiO, Ni_2O_3, NiPc, Mo foil, MoO_2 and MoO_3) were recorded in transmission mode. The spectra were processed and analyzed by the software codes Athena and Artemis.

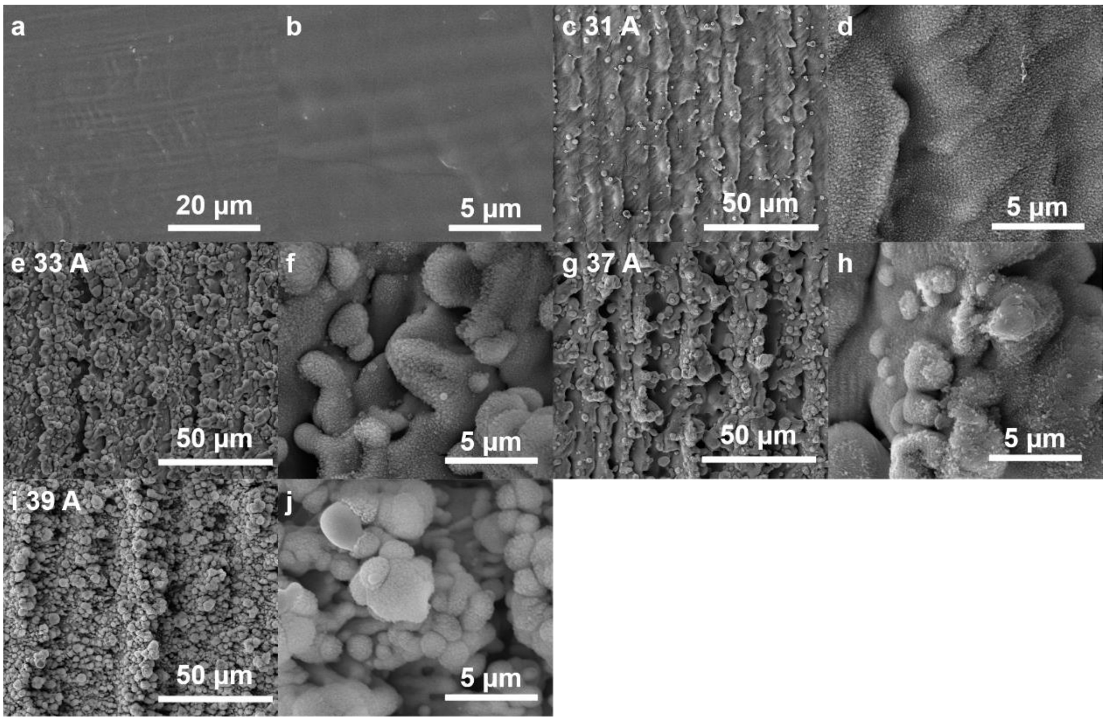

Figure S1. (a, b) SEM images of 1J85 plate. (c-i) SEM images of the 1J85-laser surfaces prepared at laser currents of 31 to 39 A.

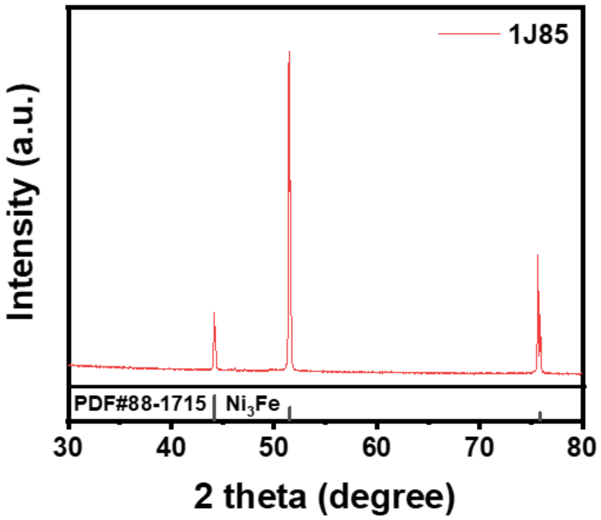

Figure S2. XRD pattern of 1J85 plate.

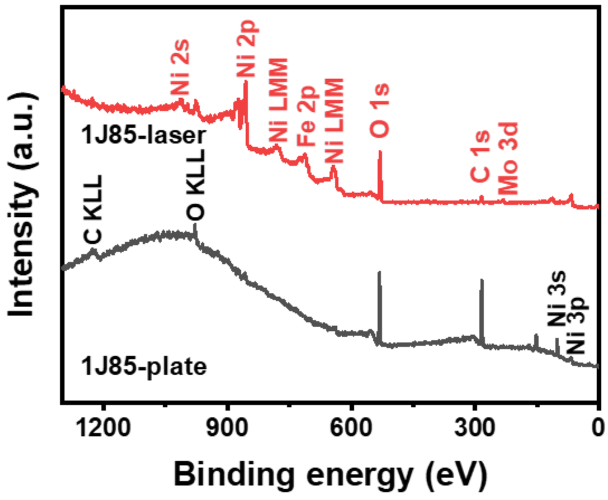

Figure S3. XPS spectra of 1J85 plate and 1J85-laser.

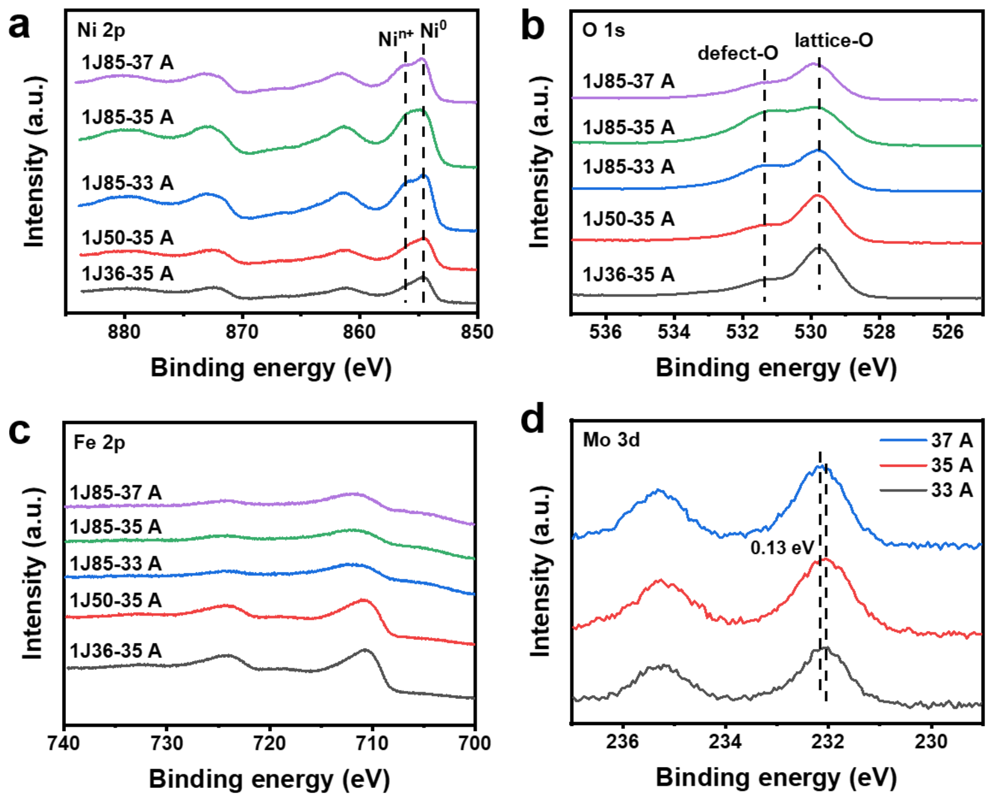

Figure S4. (a) High-resolution XPS spectra of Ni 2p for 1J36-laser, 1J50-laser, and 1J85-laser prepared by different laser currents. (b) High-resolution XPS spectra of O 1s. (c) High-resolution XPS spectra of Fe 2p. (d) High-resolution XPS spectra of Mo 3d.

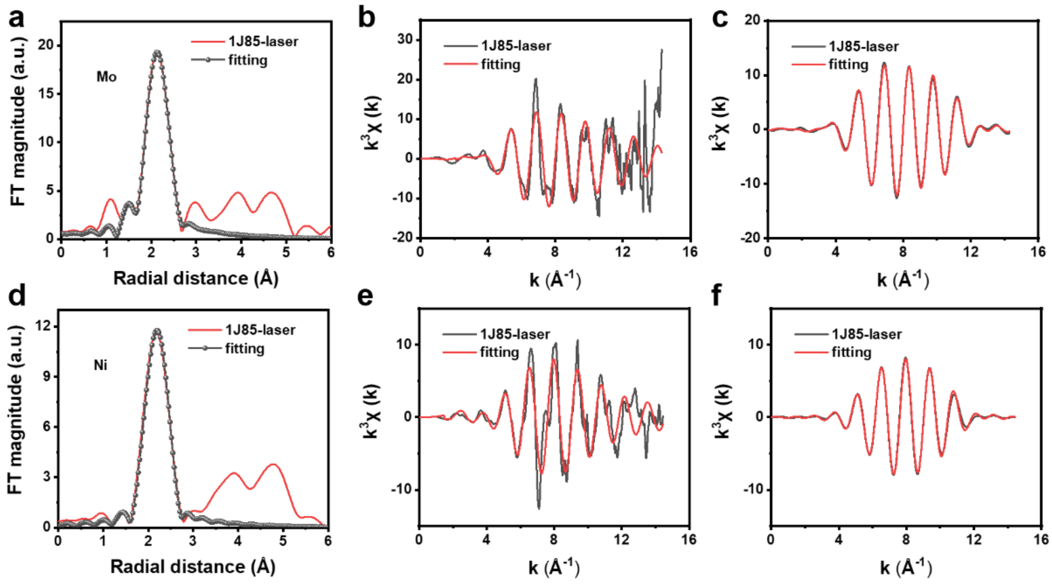

Figure S5. (a-d) Fitting curves of FT-EXAFS spectra corresponding to Figure 3b and Figure 3d. (b-e) k space fitting results. (c-f) q space fitting results.

Table S1. Structural parameters extracted from EXAFS fitting.

Sample	Edge	Path	CN	R (Å)	σ^2 (10^{-3} Å)	ΔE_0 (eV)	R-factor
1J85-laser (Mo)	Mo-K	Mo-O	0.55	2.01	1.03	16.41	0.005
		Mo-Ni	6.92	2.52	5.82	4.60	
1J85-laser (Ni)	Ni-K	Ni-Ni	4.28	2.50	6.21	8.33	0.006
		Ni-O	0.42	2.09	1.00	11.90	

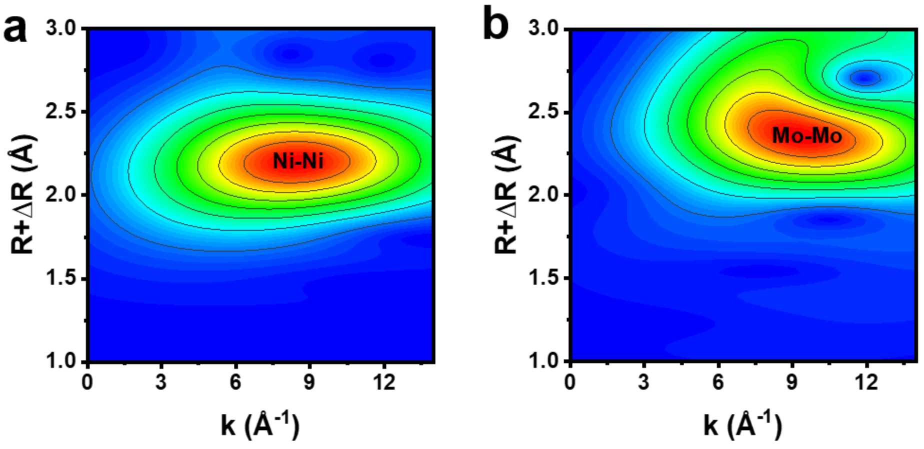

Figure S6. Wavelet transform of the Ni K-edge for (a) Ni foil and (b) Mo foil.

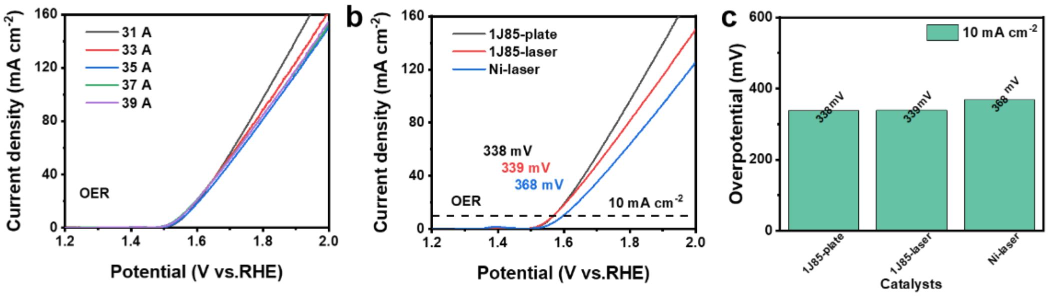

Figure S7. (a) LSV curves of 1J85-laser prepared under different laser currents in 1.0 M KOH. (b) The LSV curves of 1J85 plate, 1J85-laser, and Ni-laser in 1.0 M KOH. (c) The OER overpotential corresponding to Figure S7b.

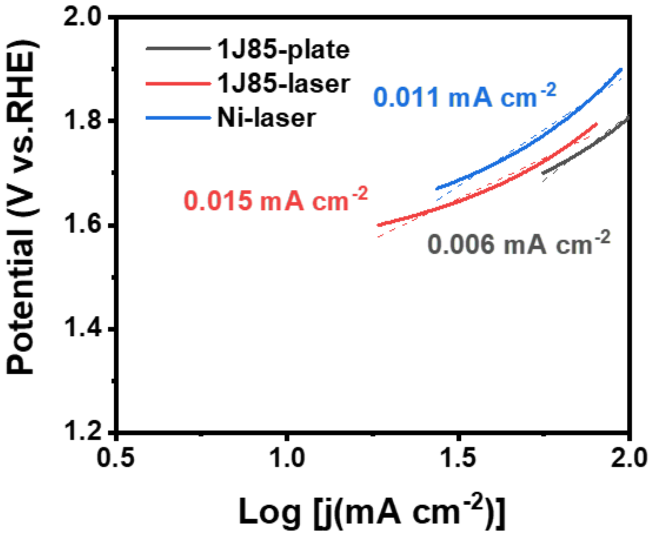

Figure S8. The exchange current density of 1J85 plate, 1J85-laser, and Ni-laser.

Table S2. Comparison HMFOR performance of 1J85-laser with other electrocatalysts reported in the paper under alkaline conditions.

Samples	Oxidation voltage (V vs RHE)	HMF Conversion (%)	FDCA Productivity (%)	HMF concentration (mM)	Reference
NiCoMn-LDHs@NF	1.50	95.0	72.9	1	[1]
$Cu(OH)_2/C$	1.45	75.8	71.2	5	[2]
$NiCo_2O_4$	1.50	90.0	84.9	5	[3]
NiOOH	1.56	96.8	86.6	5	[4]
Ni_3N-V_2O_3	1.55	/	96.1	10	[5]
CoNiFe-LDH	1.55	95.5	84.9	10	[6]
N-MoO_2/Ni_3S_2@NF	1.57	90.0	88.0	10	[7]
NiCo(-CO_3^{2-})-LDH@NF	1.56	86.0	32.0	10	[8]
1J85-laser	1.45	97.8	96.7	50	This work

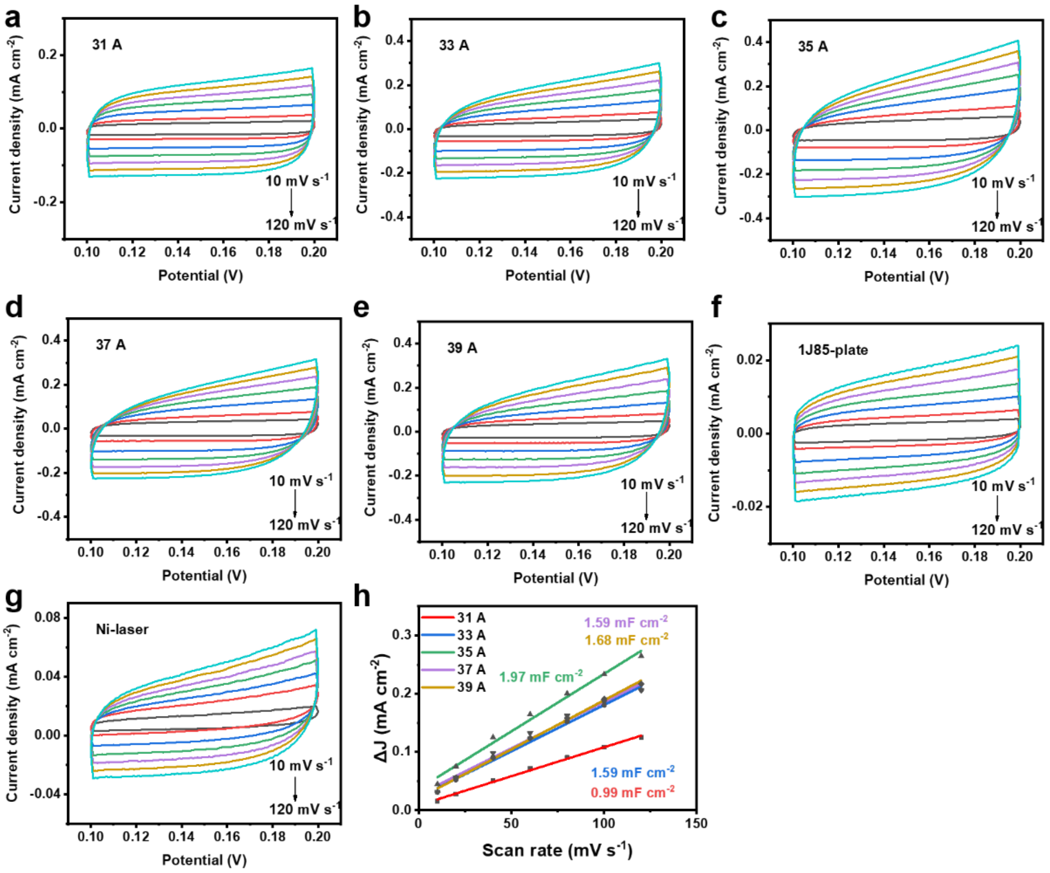

Figure S9. With scan rates from 20 to 120 mV s^{-1}, the CV curves of electrodes of (a) 1J85-laser prepared by 31 A, (b) 33 A, (c) 35 A, (d) 37 A, (e) 39 A, (f) 1J85 plate, (g) Ni-laser. (h) The corresponding capacitive current at 0.15 V as a function of the scan rate for all electrodes.

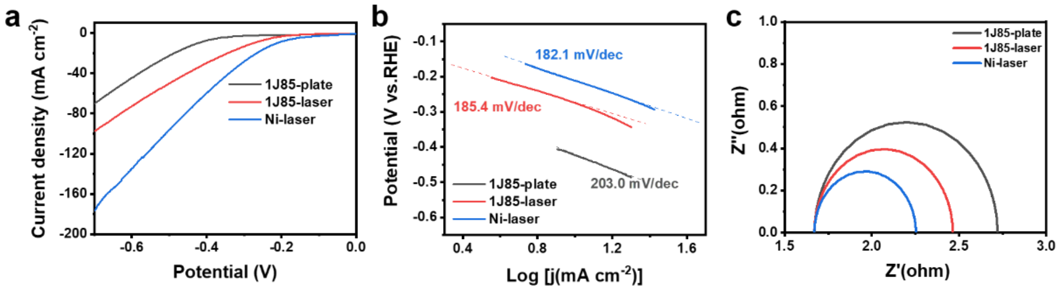

Figure S10. (a) LSV curves of electrodes to HER in 1.0 M KOH. (b) Tafel slopes corresponding to (a). (c) Nyquist plots of electrodes to HER.

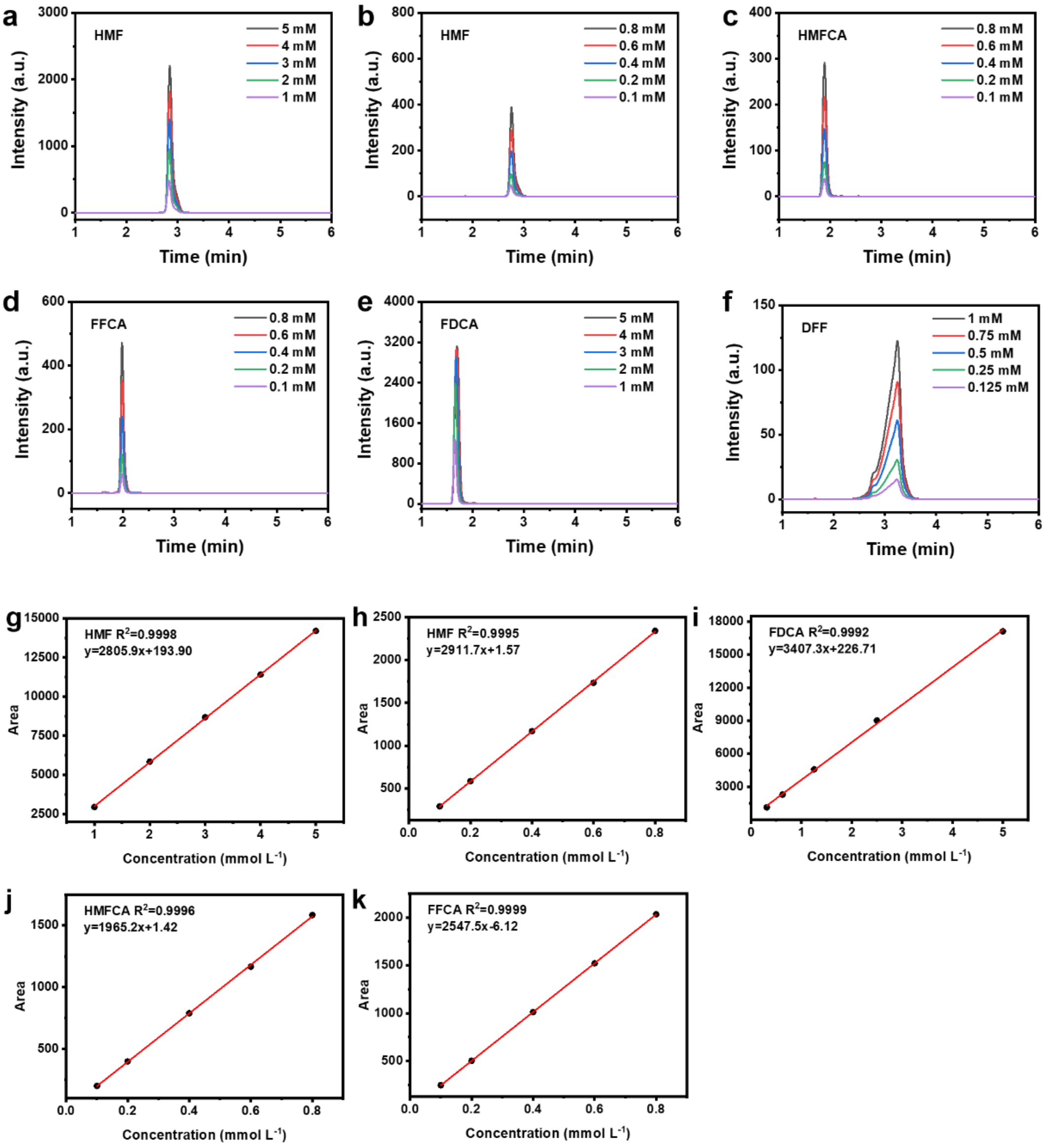

Figure S11. The LC spectra of the HMF (a-b), HMFCA (c), FFCA (d), FDCA (e), and DFF (f). External calibration curves of HMF (a-h), FDCA (i), HMFCA (j), and FFCA (k).

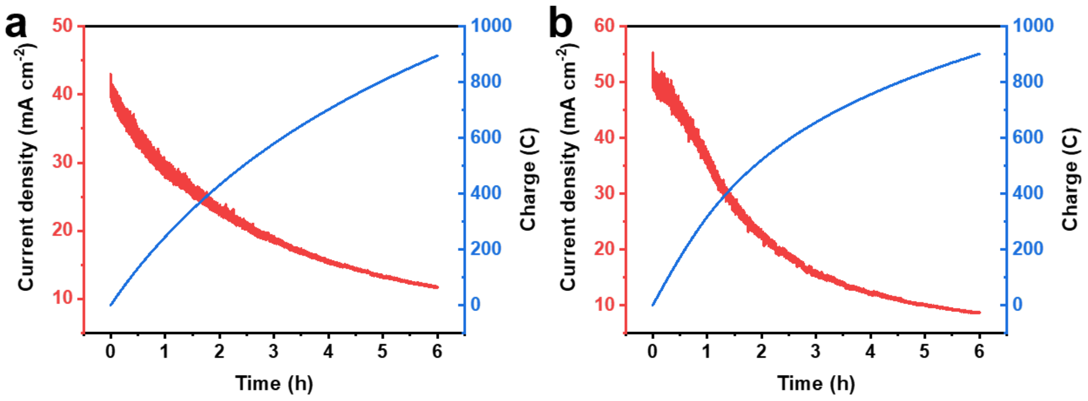

Figure S12. The i-t curves of 1J85-laser (a) and Ni-laser (b) at a constant potential of 1.55 V (vs. RHE) in 1.0 M KOH with 50 mM HMF.

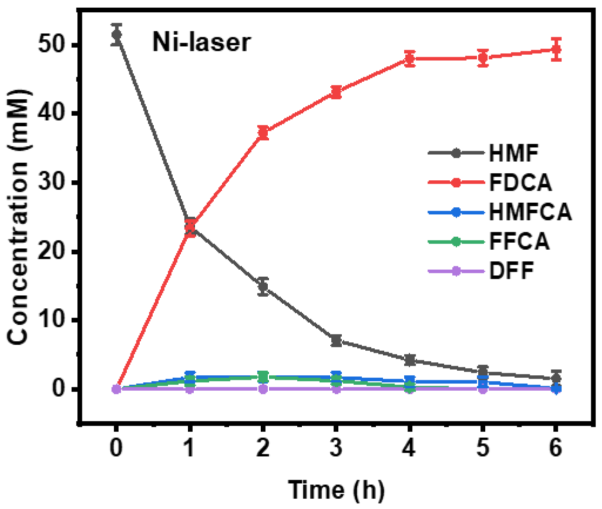

Figure S13. Curves of the concentrations of HMF, HMFCA, FFCA, FDCA, and DFF versus reaction time using Ni-laser.

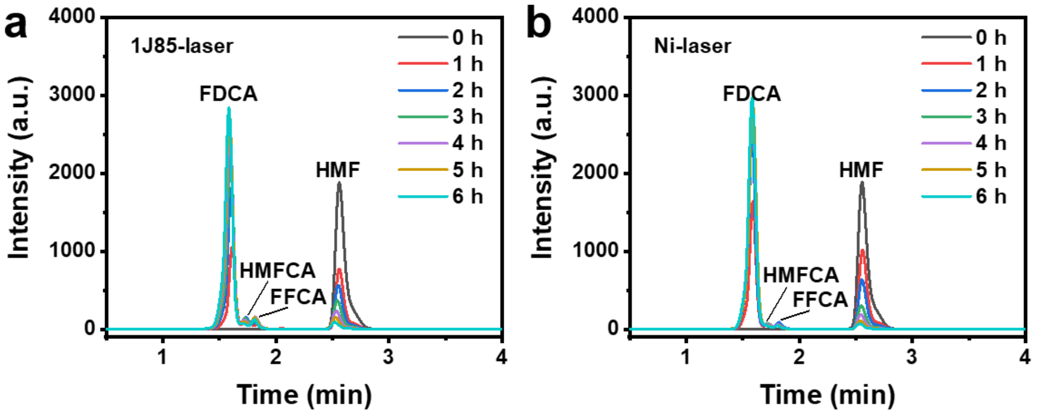

Figure S14. HPLC curves of the products obtained by electrocatalysis with 1J85-laser (a) and Ni-laser (b).

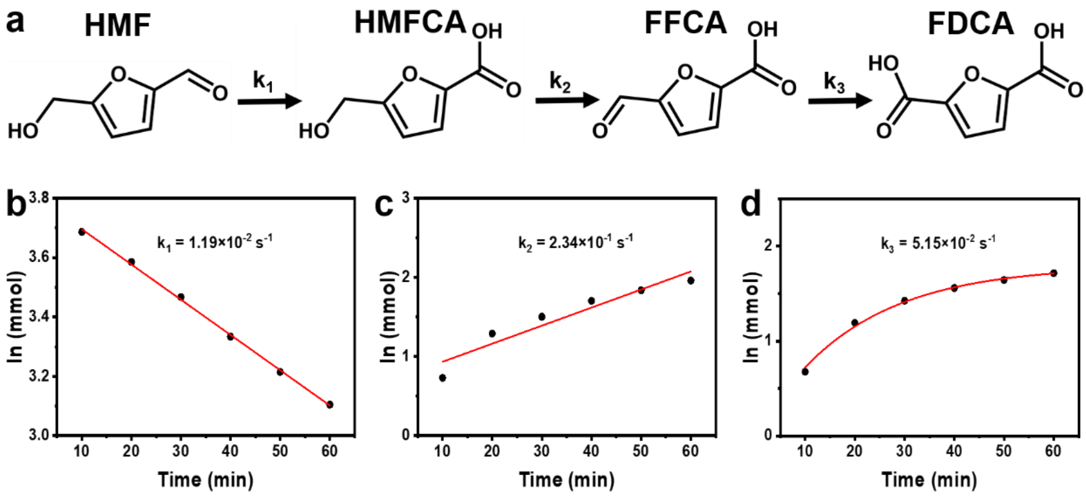

Figure S15. (a) The oxidation path of HMF with 1J85-laser. (b-d) Estimation of rate constants for each step.

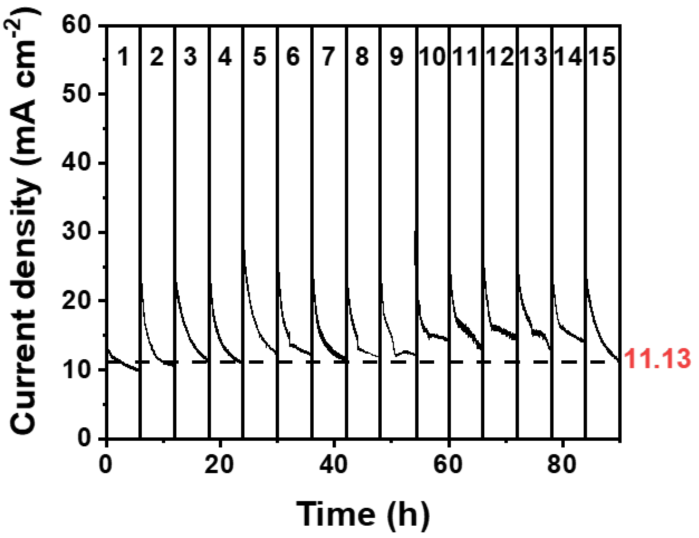

Figure S16. The i-t curves of 1J85-laser at 1.55 V with the intermittent addition of 50 mM HMF.

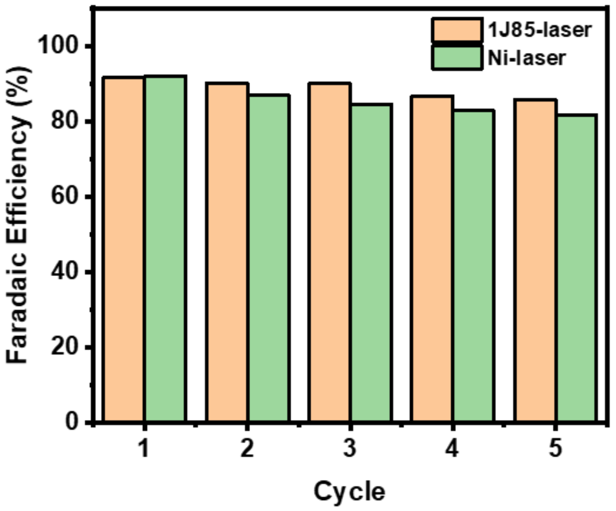

Figure S17. Faradaic efficiency of FDCA in five successive cycles with 1J85-laser and Ni-laser.

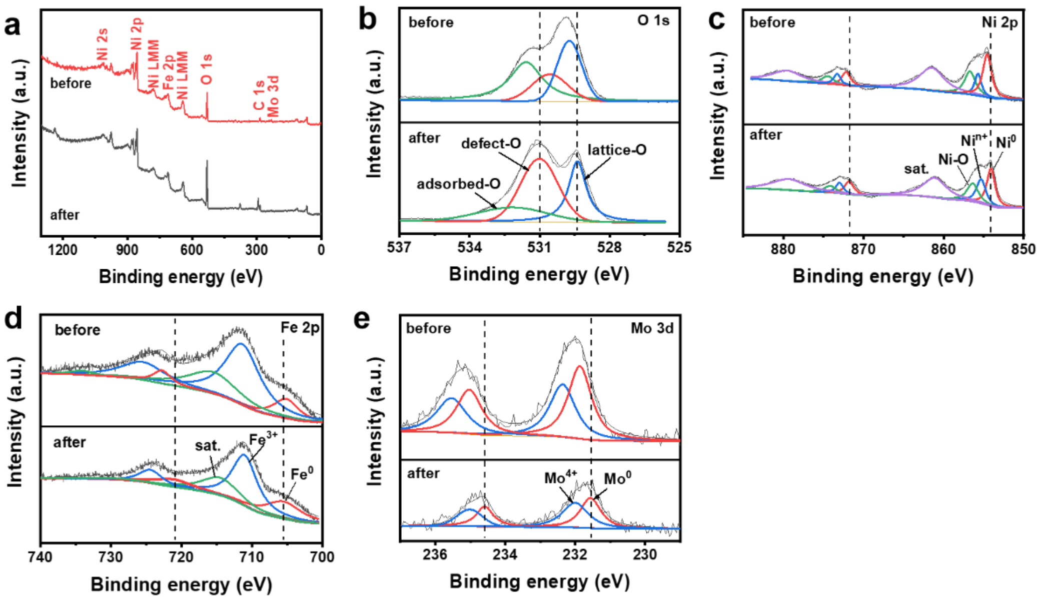

Figure S18. (a) XPS spectra of 1J85-laser before and after HMFOR. High-resolution XPS spectra of O 1s (b), Ni 2p (c), Fe 2p (d), Mo 3d (e).

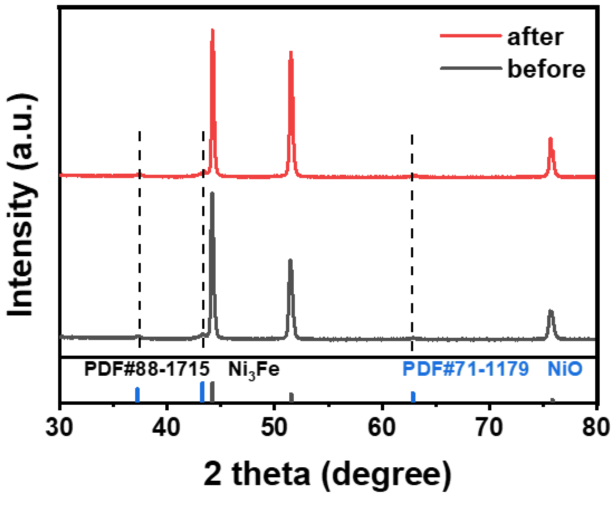

Figure S19. XRD patterns of 1J85-laser before and after HMFOR.

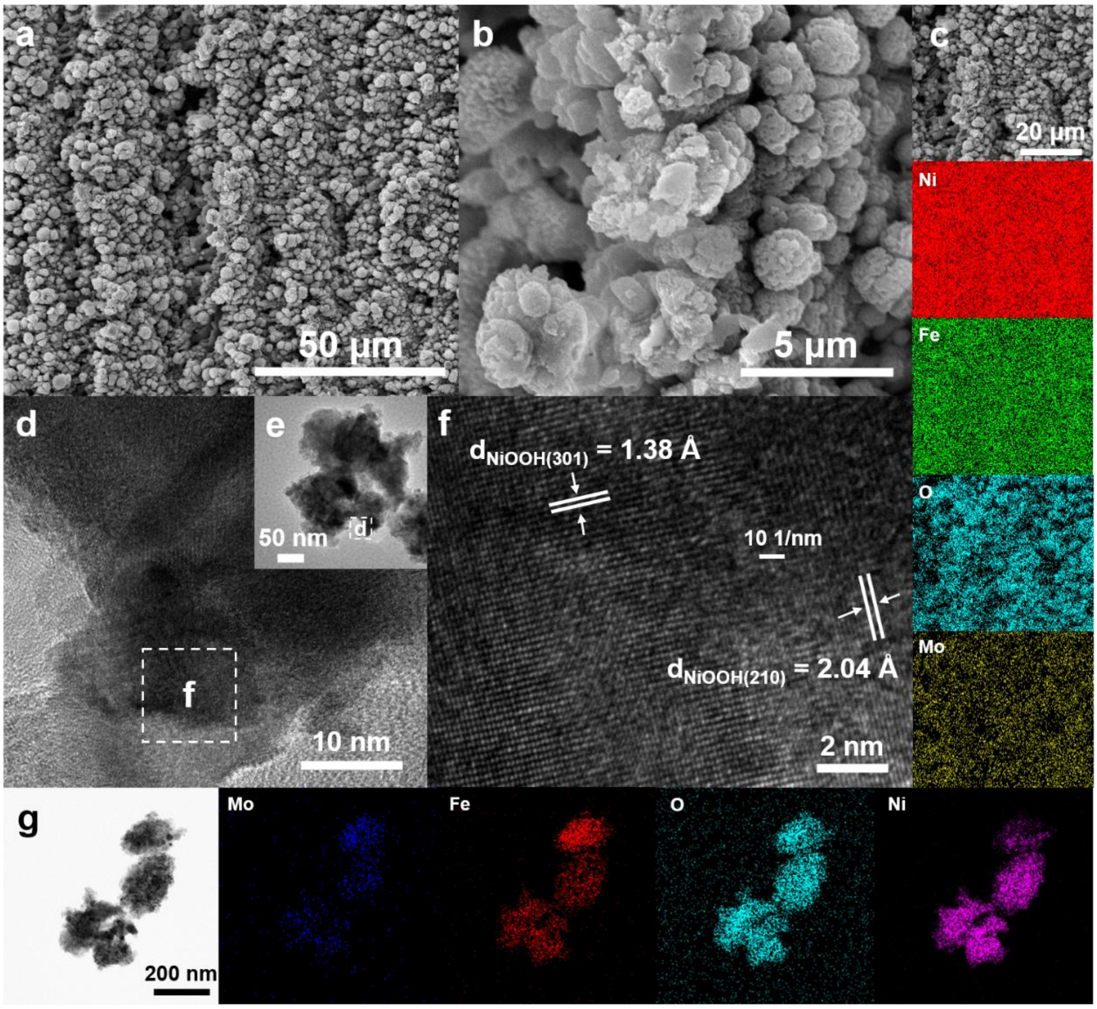

Figure S20. After HMFOR (a-b) SEM images of 1J85-laser surface. (c) SEM image of 1J85-laser and the corresponding EDS mapping images. (d, e) TEM images. (f) HRTEM image. (g) TEM elemental mapping images of 1J85-laser electrode.

Table S3. The atomic percentage of elements in 1J85-laser by TEM.

**Condition**	**element**	**Atom (%)**
Before	O K	56.21
	Ni K	36.15
	Mo L	1.26
	Fe K	6.26
	Total	100.00
After	O K	68.15
	Ni K	24.27
	Mo L	0.59
	Fe K	6.99
	Total	100.00

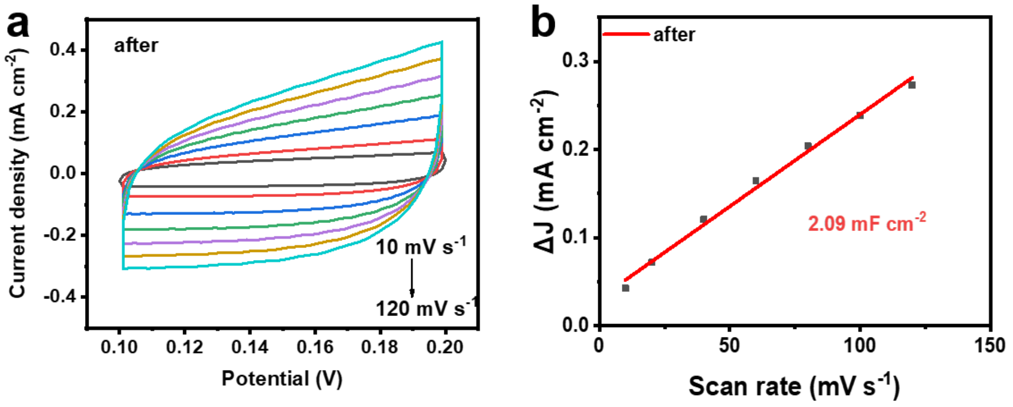

Figure S21. (a) With scan rates from 20 to 120 mV s^{-1}, the CV curves of 1J85-laser after HMFOR. (b) The corresponding capacitive current at 0.15 V as a function of the scan rate.

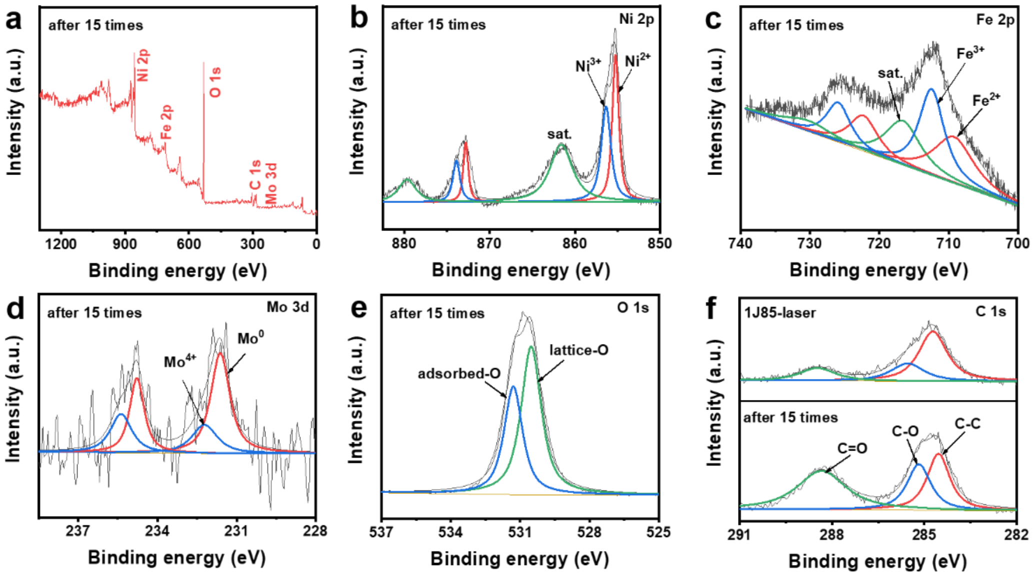

Figure S22. (a) XPS spectra of 1J85-laser after 15 times catalytic reactions. High-resolution XPS spectra of Ni 2p (b), Fe 2p (c), Mo 3d (d), O 1s (e) and C 1s (f).

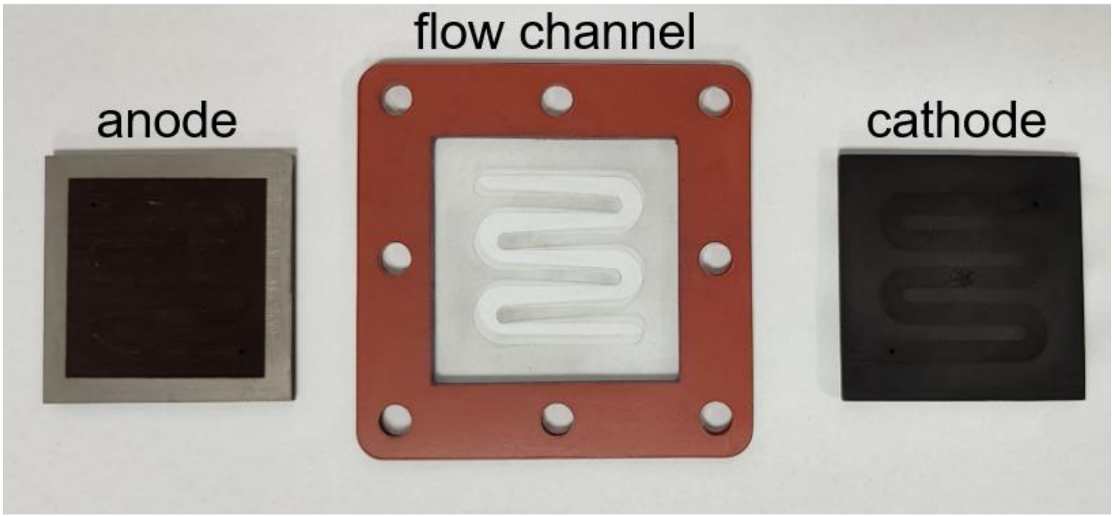

Figure S23. Photos of the anode 1J85-laser electrode, cathode graphite electrode and serpentine flow path.

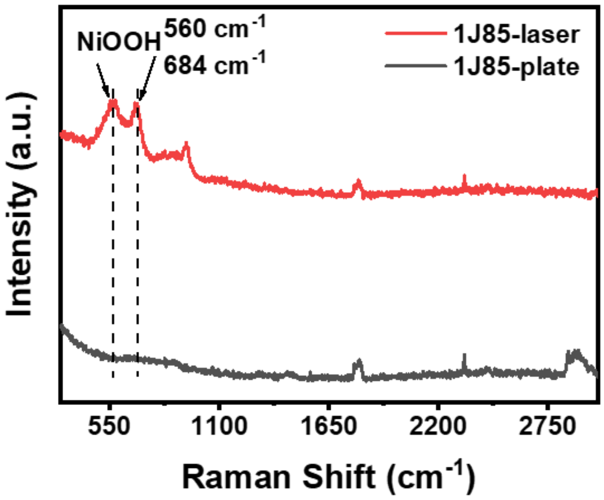

Figure S24. Raman spectra of 1J85 plate and 1J85-laser.

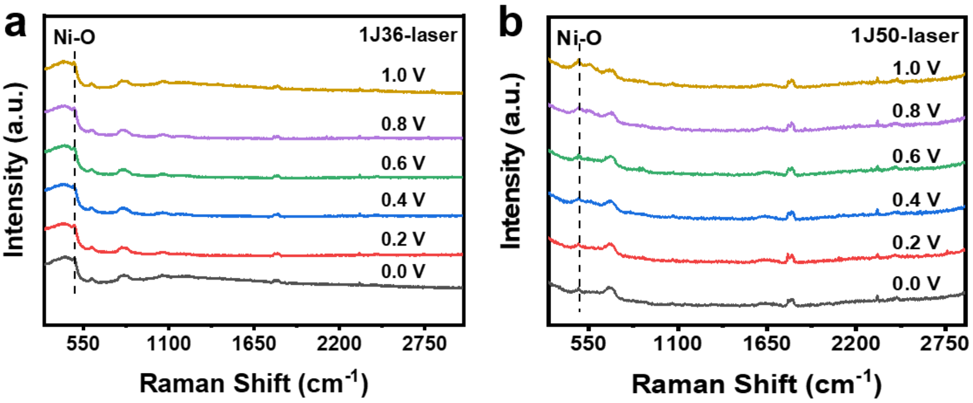

Figure S25. Electrochemical in-situ Raman spectra of 1J36-laser (a) and 1J50-laser (b) at different potentials in 1.0 M KOH with 50 mM HMF.

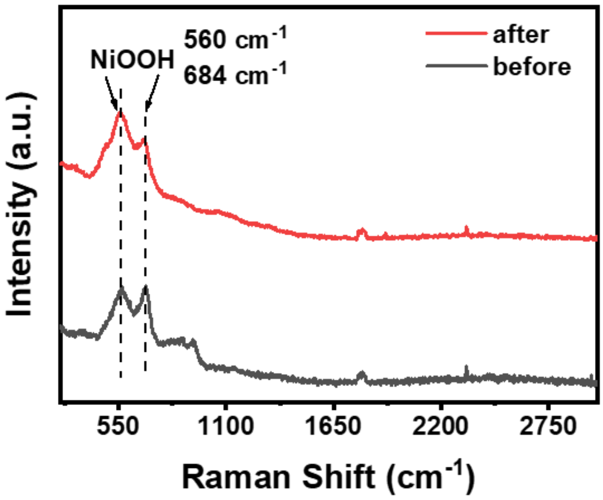

Figure S26. Raman spectra of 1J85-laser before and after HMFOR.

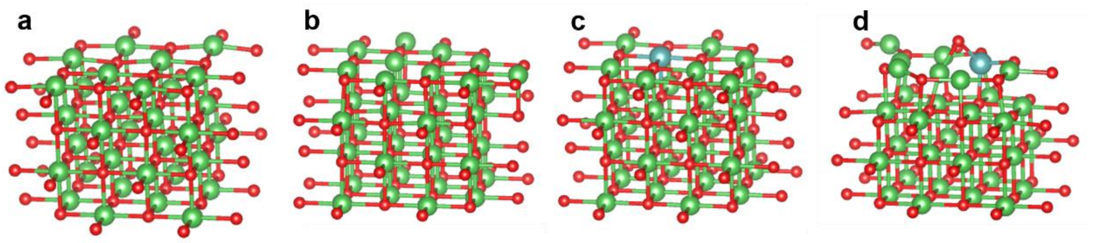

Figure S27. (a, b) Oxygen vacancy model of NiO crystal plane (200). (c, d) Oxygen vacancy model of Mo-doped NiO crystal plane (200).

References

[1] B. Liu, S. Xu, M. Zhang, X. Li, D. Decarolis, Y. Liu, Y. Wang, E.K. Gibson, C.R.A. Catlow, K. Yan, Green Chem. **2021**, 23, 4034-4043.

[2] H. Chen, J. Wang, Y. Yao, Z. Zhang, Z. Yang, J. Li, K. Chen, X. Lu, P. Ouyang, J. Fu, ChemElectroChem **2019**, 6, 5797-5801.

[3] M.J. Kang, H. Park, J. Jegal, S.Y. Hwang, Y.S. Kang, H.G. Cha, Appl. Catal. B-Environ. **2019**, 242, 85-91.

[4] B.J. Taitt, D.-H. Nam, K.-S. Choi, ACS Catal. **2018**, 9, 660-670.

[5] S. Liang, L. Pan, T. Thomas, B. Zhu, C. Chen, J. Zhang, H. Shen, J. Liu, M. Yang, Chem. Eng. J. **2021**, 415, 128864.

[6] M. Zhang, Y. Liu, B. Liu, Z. Chen, H. Xu, K. Yan, ACS Catal. **2020**, 10, 5179-5189.

[7] L. Wang, J. Cao, C. Lei, Q. Dai, B. Yang, Z. Li, X. Zhang, C. Yuan, L. Lei, Y. Hou, ACS Appl. Mater. Interfaces **2019**, 11, 27743-27750.

[8] P. Hauke, M. Klingenhof, X. Wang, J.F. de Araújo, P. Strasser, Cell Rep. Phys. Sci. **2021**, 2, 100650.
